# The Combined Expression Patterns of Ikaros Isoforms Characterize Different Hematological Tumor Subtypes

**DOI:** 10.1371/journal.pone.0082411

**Published:** 2013-12-06

**Authors:** Carlos A. Orozco, Andrés Acevedo, Lazaro Cortina, Gina E. Cuellar, Mónica Duarte, Liliana Martín, Néstor M. Mesa, Javier Muñoz, Carlos A. Portilla, Sandra M. Quijano, Guillermo Quintero, Miriam Rodriguez, Carlos E. Saavedra, Helena Groot, María M. Torres, Valeriano López-Segura

**Affiliations:** 1 Laboratory of Human Genetics, Los Andes University, Bogotá, Colombia; 2 Pathology and Laboratories, Santa Fe de Bogotá Fundation, Bogotá, Colombia; 3 Pediatric Hemato-Oncology Unit, University Hospital del Valle, Cali, Colombia; Bellvitge Biomedical Research Institute (IDIBELL), Spain

## Abstract

A variety of genetic alterations are considered hallmarks of cancer development and progression. The Ikaros gene family, encoding for key transcription factors in hematopoietic development, provides several examples as genetic defects in these genes are associated with the development of different types of leukemia. However, the complex patterns of expression of isoforms in Ikaros family genes has prevented their use as clinical markers.

In this study, we propose the use of the expression profiles of the Ikaros isoforms to classify various hematological tumor diseases. We have standardized a quantitative PCR protocol to estimate the expression levels of the Ikaros gene exons. Our analysis reveals that these levels are associated with specific types of leukemia and we have found differences in the levels of expression relative to five interexonic Ikaros regions for all diseases studied. In conclusion, our method has allowed us to precisely discriminate between B-ALL, CLL and MM cases. Differences between the groups of lymphoid and myeloid pathologies were also identified in the same way.

## Introduction

The Ikaros family includes five transcription factors (Ikaros, Helios, Aiolos, Eos and Pegasus) with a key role in the regulation of the hematopoietic system by both potentiating and repressing gene expression [1-3]. All members share the same structure with two functional domains composed of zinc fingers [4]. The first four zinc fingers encode for a DNA-binding domain (DBD), whereas the last two encode for a dimerization domain, which allows the binding between isoforms of the family [5]. These domains are encoded by different exons and depending on the splicing, different isoforms can be generated with lack of certain zinc fingers [6-10]. Isoforms that display less than three zinc fingers in the DBD are considered dominant negative (DN) as not only they are defective but also may interfere with the activity of functional isoforms [11].

 Over the last decade, several studies have related defects in different members of this family of transcription factors with the development of hematopoietic neoplasms. Early studies on these genes revealed the expression of different DN isoforms of Ikaros (Ik-4, Ik-7 and Ik-8), as well as insertions of 60 bp in Exon 2 and deletions of 30 bp in Exon 6, in infants diagnosed with ALL [12-14]. Another DN, Ik-6, has been found to be over-expressed in patients with different hematological diseases, including B Acute Lymphoblastic Leukemia (B-ALL), T Acute lymphoblastic Leukemia (T-ALL), Acute Myeloid Leukemia (AML), Chronic Myeloid Leukemia (CML), and is one of the isoforms with the highest prevalence in several types of hematological neoplasms [15-19]. 

 Similarly, the DN variants of Ikaros related genes, such as Helios and Aiolos, have been associated with various types of leukemia. Studies of Helios conducted in cell lines derived from human lineage T leukemia and lymphomas showed an over-expression of the DN He-5 and He-6 isoforms, which lack the first DBD zinc fingers. These isoforms, along with the DN He-7 and He-8, have been found in patients with T-ALL [20,21]. In addition, a new isoform mistakenly called He-3, which lacks the entire N-terminal end has been described in cell lines derived from B and T ALL [22].

 In the case of Aiolos, the study on null mouse models has shown an important role as a B cell-specific tumor suppressor gene. No changes for Aiolos expression have been reported in human leukemia, but interestingly it has been determined over-expression of Aiolos in several human lymphoma subtypes [23]. Aiolos has been linked to cell survival through the interaction of Bcl-xL and DT40. This could explain the higher resistance of lymphomas to apoptosis, a recurrent physiopathologic trend described for these neoplasms [24].

 It has been reported a drastic decrease of the expression of functional isoforms, such as Ik-1, Ik-2, He-1, He-2 and Aio-1 in all these studies together with the increased expression of DN isoforms [17,21]. Taking into account all these data, we must consider that over-expression of DN isoforms is not the sole physiopathological mechanism, but that the imbalance between all isoforms may have an important role in the development of the disease, especially considering that DN isoforms also appear in healthy individuals.

 Despite the knowledge about the prevalence of DN isoforms of Ikaros family genes in hematological neoplasias, there are no reported protocols to use the expression levels of these isoforms for clinical purposes, mainly because of the absence of quantitative data. Most of the aforementioned studies only correspond to qualitative analyses focusing on the presence of various isoforms in patients [15-19]. Moreover, the techniques used have been directed to the DN isoform of interest, but they are unable to provide information about the whole group of isoforms, which would be a true indicator of each pathological condition.

 The first quantitative approach for the study of the Ikaros gene was carried out by Iacobucci and collaborators using a high-performance capillary electrophoresis on cases of Philadelphia chromosome positive ALL. While this technique allowed them the quantification of different peaks corresponding to different isoforms, the most important result of such study was that 41% of patients displayed over-expression of the DN Ik-6 isoform [25], although a complete molecular profile of the expression in this family was not obtained.

Currently, there are no strategies able to accurately quantify splicing isoforms of a gene. All splicing isoforms present in a sample cannot be detected even through modern genomic techniques (es eso cierto?). Whis this in mind, we have designed a method based on qRT-PCR that allowed us to get closer to the set of isoforms existing in each pathological condition and extract credible data from the full expression of this gene in every type of disease.

## Material and Methods

### Patients and Samples

We studied 46 bone marrow samples from patients with different types of hematologic malignancy (15 B Acute Lymphoblastic Leukemia (B-ALL), 14 Acute Myeloid Leukemia, 5 Chronic Myeloid Leukemia (CML), 7 Cronic Lymphoid Leukemia (CLL) and 5 Multiple Myeloma (MM) cases, with an age range between 3 and 90 years old) (Table 1), which were obtained for diagnostic purposes at the Santa Fe de Bogotá Fundation University Hospital (Colombia) (Table 1). Cell selection was not performed, but only patients who showed a clear enrichment in specific cell types were included in the study. The study was approved by Los Andes University and Santa Fe de Bogotá Hospital ethics committees and a written informed consent form was obtained from each patient prior to sample processing. In the case of children samples, parents, caretakers or guardians signed written informed consent. All the consents of the samples to study are in custody of the principal investigator of the work.

**Table 1:** Clinical and Patient Characteristics.

Pathology	Number of Samples	Age mean (min./max.)
Acute myeloid leukemia (AML)	14	62,3 (23/90)
B Acute lymphoblastic leukemia (B-ALL)	15	16,42 (3/42)
Chronic Myeloid Leukemia (CML)	5	58,5 (41/76)
Chronic lymphocytic leukemia (CLL)	7	73 (72/74)
Multiple-Myeloma (MM)	5	65 (41/78)

All samples were characterized by morphology and flow cytometry analysis. We used blood samples from three healthy individuals (8, 22 and 64 years old) as controls.

### RNA Extraction and cDNA Synthesis

We extracted total RNA from bone marrow samples with TRIzol reagent (Invitrogen) following the manufacturer's instructions. To do this, we used 500 μL of TRIzol and 200 μL of the sample. The RNA was resuspended in distilled-deionized water (ddH2O) and was stored at -80°C. The quality and integrity of RNA was analyzed by spectrophotometry on a Nanodrop (Thermo Scientific). Only samples with values between 1.8-2.1 of OD260/OD280 ratio were used for reverse transcription process. RNA integrity was checked by electrophoresis in 1% agarose gel under denaturing conditions. The two subunits of ribosomal RNA—18s and 28s—were visualized for each sample.

cDNA was synthesized using the reverse transcriptase kit IMPROM II (Promega) according to manufacturer specifications. 1 μg of RNA was used for cDNA synthesis. The cDNA was quantified by spectrophotometry on a Nanodrop (Thermo Cientific). The resulting cDNA was stored at -80°C until use.

### Quantitative PCR (qRT-PCR) Standardization

Several Ikaros isoforms previously cloned in plasmids were used for the standardization of the qRT-PCRs and for the construction of the standard curves. Appropriate efficiencies between the range of 90 -110% (R2=0.996) were obtained using serial dilutions (25, 50, 100 and 200 ng) of the previous obtained isoforms to confirm the reproducibility of our data.

The qRT-PCRs efficiency was also monitored in every experiment using standard curves from the different PCRs randomly. This efficiency and the normalizing gene values were calculated in each of the assemblies of qRT-PCR and these data were integrated into the software qbasePLUS. To assess the specificity of the primers and the qRT-PCR, we studied the melting curve of each Amplicon with cloned material, which proved that the qRT-PCR was specific (Data not shown).

The PCRs were performed with primers designed in interexonic region 2-3 (Ik 2-3F 5’-GATCCCCGAGGACCTCTC-3’, Ik2-3R 5’-CGTAAATCCTCCGCACATTC-3’), Interexon 3-4 (Ik3-4F 5’-TGTGATATCTGTGGGATCATTTG-3’, IK3-4R 5’-GGAATGCAGCTTGATGTGC-3’), interexon 4-5 (IK4-5F 5’-CCCTTCAAATGCCACCTCT-3’, IK4-5R 5’-GCAGCGCTCTTTATGTTCCT-3’), interexon 5-6 (IK5-6F 5’-AGAGCGCTGCCACAACTACT-3’, IK5-6R 5’-GGCGACGTTACTTGCTAGTCT-3’) and interexon 6-7 (IK6-7F 5’-AGCAAGTAACGTCGCCAAAC-3’, IK6-7R 5’-CGTTGTTGATGGCTTGGTC-3’). To achieve this, we designed forward primers at the end, and reverse primers at the beginning of each exon. Interexon 1-2 was not taken into account because in this region there are no splicing variants reported. These primers were designed on the Ikaros sequences BT009836.1 taken from the GenBank of The National Center for Biotechnology Information (NCBI). Some of the parameters such as the affinity of our primers for the target sequence, annealing temperatures and specificity between each other were assessed by the web program IDT Scitools PrimerQuest SM (www.idtADN.com).

The minimum information for publication of quantitative Real-time PCR experiments (MIQE) standards were used for the design of the qRT-PCR experiments [26-28]. The primers were designed to amplify regions between 75 and 150 bp, with a 50-60% GC content and a theoretical annealing temperature (Tm) between 55 and 65°C. The glyceraldehyde 3-phosphate dehydrogenase gene (GAPDH) was used for the normalization of the qRT-PCR. The mean threshold cycle (Ct) for the GAPDH was 27.42±1.35, a very robust data for an internal control.

The experiments were conducted with an IQ-5 thermal cycler (Biorad^TM^). Detection of the amplicons was evaluated with the SYBR Green intercalant (SsoFast^TM^ EvaGreen^®^ Supermix, Biorad^TM^). The standard protocol was conducted according to the manufacturer instructions as follows: activation of the enzyme at 95 °C for 30 sec, denaturation at 95 °C for 5 sec, and annealing at 55 °C for 10 sec. The melting curve was standardized with the following parameters: temperature range 65-95°C with the temperature increasing in 0.5°C increments and measurements in 3 minute intervals. 

### Statistical Analyses

The quantification and standardization of results were conducted using the software qbasePLUS version 2.1vb (Biogazelle), which allowed us to perform a ΔΔCt quantification with a modification to the formula that enables the integration of data for the efficiencies of target and reference genes. This allows for more accurate gene expression analysis.

Statistical tests were conducted with the "R" statistical package version 2.11.1. The assessment of data normality was determined by the Shapiro-Wilk test, followed by the randomization (5,000 iterations) of our data using the ImPerm package of "R". We then performed a multi-factor ANOVA, and pairs were finally analyzed using the Tukey test.

Hierarchical clustering analysis was performed using standard correlation coefficients; for the cluster method, a similarity metric and centroid linkage were used. Briefly, the MFI value obtained for each PCR tested was introduced into a dedicated database system (Microsoft Excel, Microsoft, Seattle, WA). A ratio between the MFI obtained for each PCR in cases and the mean MFI value for three controls was then calculated. Clustering was performed after median centering and normalizing the fluorescence ratios. A logarithmic (base 2) transformation was applied to the values of this ratio for individual data sets. The resulting normalized log 2 ratios were used for further statistical analysis with the Cluster 3.0 (C Clustering Library 1.5) and Java Treeview 1.1.6r2 programs [29].

## Results

### The Expression of Ikaros is Different in Each Group of Diseases

We first obtained the expression details for each sample and interexon (Ct), and subjected the results to the statistical analysis of normality using the Shapiro-Wilks test. The results obtained from many pathologies showed that the data do not follow a normal distribution. Taking this into account and due to the differences in the number of samples between each of the groups of pathologies, we performed a randomization of data (5,000 iterations) using imPerm package of the programme "R" followed by multifactorial ANOVA (Table 2). The results of this analysis showed that there are significant differences between all the groups of pathologies studied regarding the level of expression of the various interexons, understanding significance in each group as the statistical difference found at least between a pair of pathologies concerning the values of expression in every interexon.

**Table 2:** ANOVA Results.

Interexon	Df	Sum Sq	Mean Sq	F value	PrPr(F)
2-3	4	2.1982	0.54955	5000	2e-04 ***
3-4	4	2.1844	0.54611	5000	6e-04 ***
4-5	4	69.551	17.3877	5000	< 2.2e-16***
5-6	4	9.0364	2.25909	5000	< 2.2e-16***
6-7	4	78.672	19.6680	5000	< 2.2e-16***

*=P< 0.05

^**^ =P< 0.01

^***^ =P< 0.001

**Figure 1:**
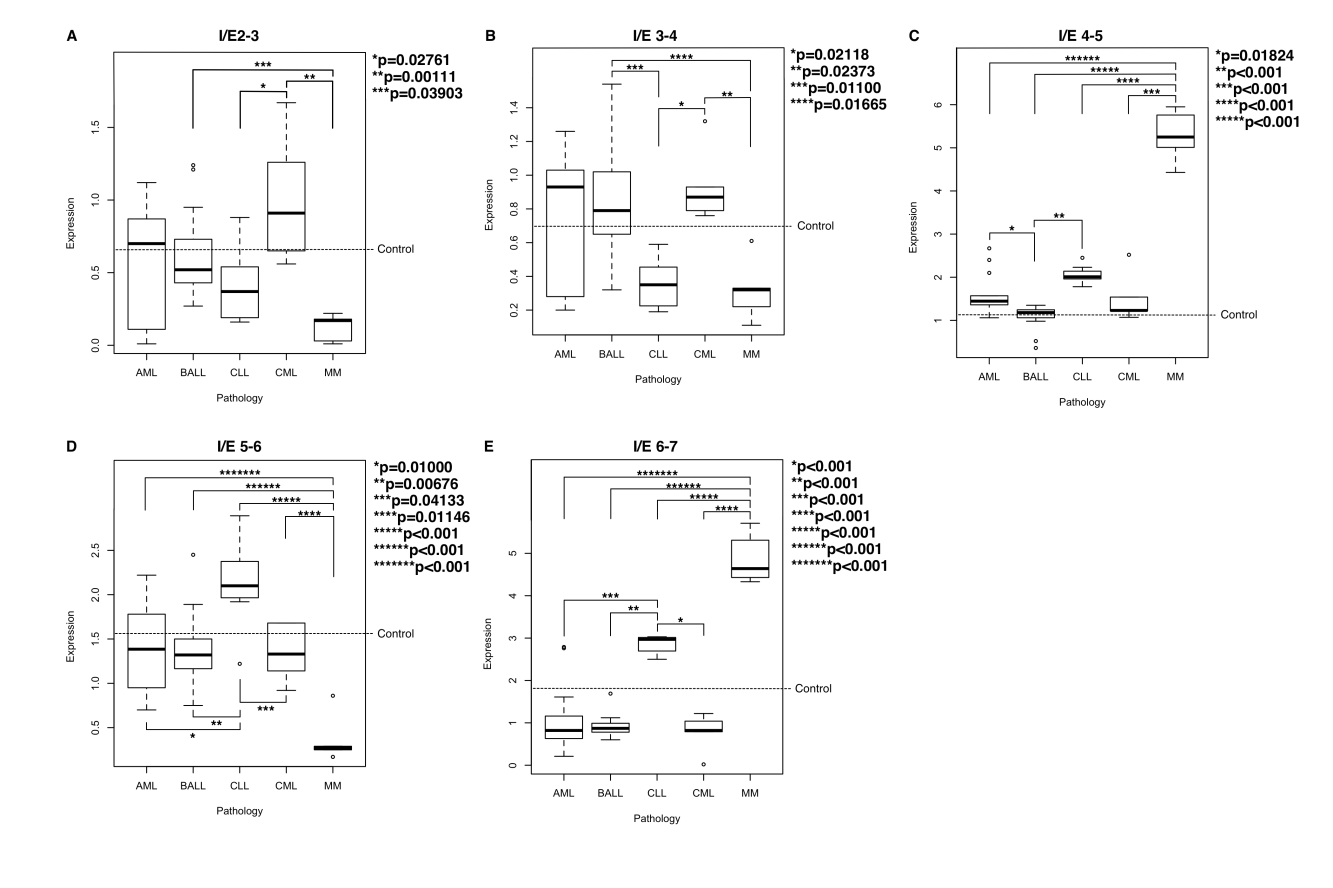
Level of expression of every interexon (I/E) in the assessed pathologies. (A) expression of the interexon 2-3, (B) expression of the interexon 3-4, (C) expression of interexon 4-5, (D) expression of the inter-exon 56 and (E) expression of interexon 6-7. CML (chronic myeloid leukemia), MM (multiple myeloma), CLL (chronic lymphoid leukemia), BALL ( B acute Lymphoblastic leukemia), AML (acute myeloid leukemia). The control line is the arithmetic media (s.d. <0,01 in all PCR) of three healthy peripherial Blood controls.

Once we obtained the significance values, we used the Tukey test to establish differences among the groups of pathologies. The results showed that all pathologies present differences in some interexon evaluated, as predicted by the ANOVA test (the graphic interpretation of these data are presented in Figure 1).

The differences were more prominent for some of them, in which a single exon allowed us to identify the nature of some of the pathologies. For the expression of the interexon 2-3 (Figure 1A), there are highly significant differences between the CML compared to the other groups, like MM and CLL. This seems to indicate that the higher expression of this region is something characteristic of this type of leukemia, whereas the rest of the pathologies displayed a lower expression value. The very low expression levels in the case of MM enables the possibility of discriminating between this pathology from CML and ALL with a very high significance. In the case of interexon 3-4 (Figure 1B), all groups repeat a pattern similar to the one observed in interexon 2-3 with a tendency for overexpression for the CML group, but with less differences against AML and B-ALL. Unlike what is observed for the interexon 2-3, in this interexon there are differences of CLL with CML and, in this case, with B-ALL. The study of interexon 4-5 (Figure 1C) showed significant differences between many of the groups, allowing us to discriminate the MM group directly against all other groups. These differences result from the low expression of this amplicon in all the other pathologies. The high capacity to discriminate between these pathologies, without any overlap and small deviation, led us to the conclusion that it may be used as a biomarker for this pathology. The importance of this interexon should be noted because this region code for several zinc fingers of the DBD. There are also differences between B-ALL and CLL and between B-ALL and AML. In the case of the interexon 5-6 (Figure 1D), there is a difference between CLL and the other groups. Similarly, there are very strong differences between MM and all the others. Finally, the interexon 6-7 (Figure 1E) repeats the pattern observed in Figure 1C. All groups show differences with respect to MM. In addition, this interexon also allows us to differentiate the CLL from the rest of the pathologies. In all cases, stable expression was found, and over-expression in CLL and MM showed that the majority of isoforms contained the carboxyl-terminal end in these two pathologies. Note the high discriminatory capacity of this interexon, with p<0.001 in all comparisons and minimum standard deviations for all diseases.

### Detection of Variants of Splicing by Melting Curve

The presence of two or more peaks in an amplicon melting curve gives us information about the existence of a non-canonical splicing, in which certain insertions or deletions could be expected. In this way, we detected specific splicing variants in the interexon 4-5 and interexon 6-7 in specific diseases (Table 3), which can be a distinctive feature for these groups of pathologies. In this regard, there appears to be a relationship between the isoforms with no canonical splicing variants (two melting peaks at 84 and 86,5°C) in the 4-5 interexon and B-ALL, in which 86,67% of cases showed two melting peaks. This non-canonical splicing are present in CLL and MM too, which seems to indicate that it is a characteristic of the lymphoid diseases only. For the interexon 6-7, we found the same anomaly with two peaks at 82 and 85 °C on the melting curve represented in some cases of B-ALL. While the percentage is much lower (33,3%), the presence of non-canonical splicing is specific to this pathology, and does not appear in any other type of disorder. This seems to show that in the others diseases there is no non-canonical splicing and alternative isoforms are only obtained by exons alternation in this region. In addition, this could indicate the existence of molecular B-ALL subcategories.

**Table 3:** Study of Non-Canonical Splincing through the Melting Temperature.

Pathology	Cases number	# 2 peaks interexon 4-5	Tª(°C)	# 1 peak interexon 4-5	Tª(°C)	# 2 peaks interexon 6-7	Tª(°C)	# 1 peak interexon 6-7	Tª(°C)
B-ALL	15	13 (86,67%)	84/86,5	2	86,5	5 (33,3%)	82/85	10	85
CLL	7	4(57,14%)	84/86	3	86,5	0	85	8	85
MM	5	3(60%)	84/86,5	2	86,5	0	85	5	85
AML	14	0	86,5	16	86,5	0	85	16	85
CML	5	0	86,5	5	86,5	0	85	4	85

### The Ikaros Profile

**Figure 2:**
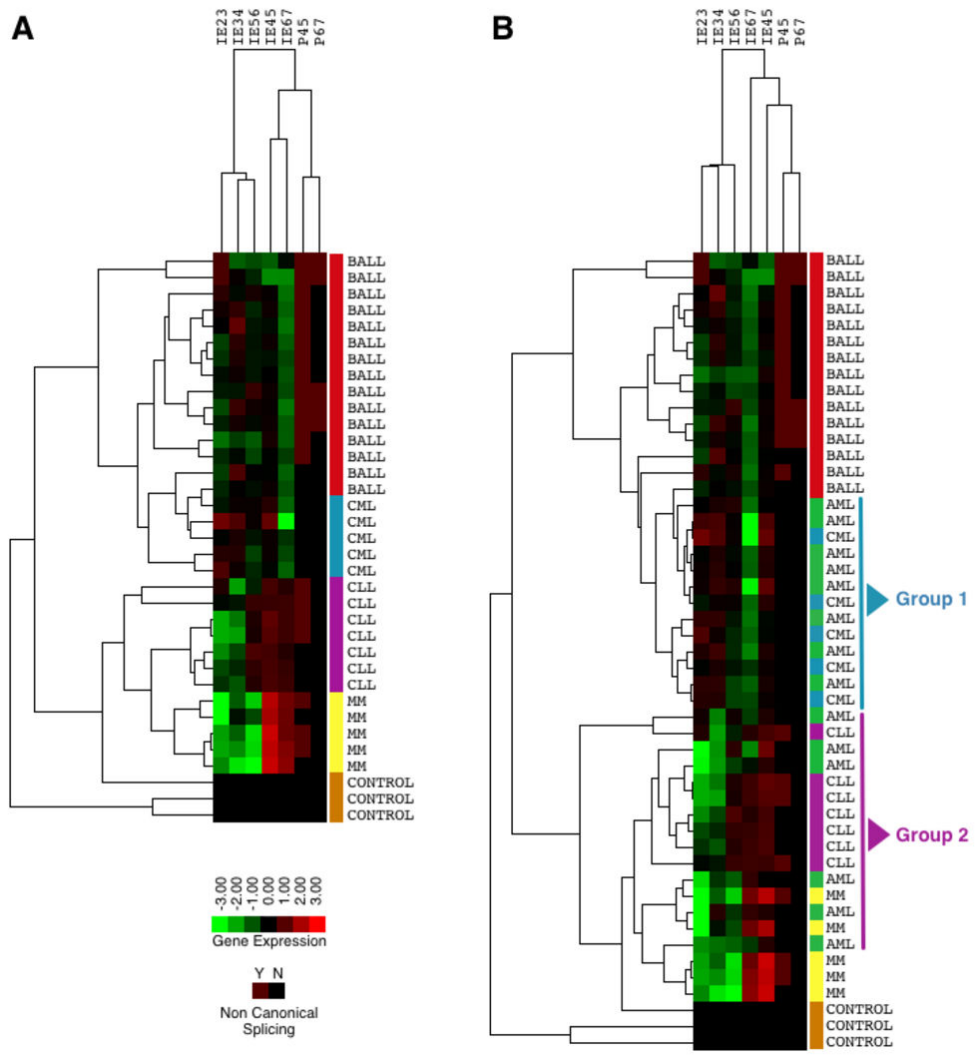
Hierarchical cluster analysis of exon differentially expressed in B-ALL, CLL, CML, AML and MM cases. Mean fluorescence intensity (MFI) values obtained for each PCR were imported and normalized according to mean MFI value observed for that PCR in control cases. Rows represent the B-ALL, CLL, CML, MM cases, while columns correspond to the normalized log_2_ ratios of the MFI values obtained for each inter-exon PCR analyzed. The relative level of expression of each PCR is represented in a color code: red represents expression greater than the mean, green represents expression lower than the mean, and the color intensity represents the magnitude of the deviation from the mean; the scale extends from ratios of -3 to +3 (log_2_ units). P4-5 and p6-7 columns represent the presence (Y) or absence (N) of non canonical splincing. (A) Cluster without the heterogeneous group of AML, (B) Cluster of all pathologies.

To obtain a complete profile of the expression of Ikaros isoforms, we analyzed the information for all the interexons by a hierarchical clustering of the pathologies. The clustering allowed us to observe that all the groups except AML were homogeneous. This group cannot be separated from the rest and share similarities with CLL and CML (Figure 2B). In fact, two subgroups can be distinguished, the first group with similarity to the CML and a second group closer to the CLL. When we performed the analysis of these two groups by the Tukey test, we observed that they differ depending on the levels of expression of the interexons 2-3, 3-4 and 6-7 with a highly significant p value (Figure S1 and S2). 

When only B-ALL, CML, CLL and MM groups were compared, we could separate the four groups of pathologies (Figure 2A). The B-ALL and CML pathologies are characterized by a very low expression of interexon 6-7. In the case of the B-ALL, it is also important to highlight the presence of non-canonical splicing very characteristic of this disease. All CLL cases have a higher expression of the last three interexons than the first two. In this case, samples could be divided with respect to the presence of non-canonical splicing. Finally, the MM cases, as one would expect show a very representative profile with a high overexpression of interexons 4-5 and 6-7 and also a marked low expression of the other interexons (Figure 2A). 

### Differences in the Expression of Ikaros between Lymphoid and Myeloid Disorders

Ikaros is an important regulator of gene expression playing a very important role in the differentiation, proliferation and lymphoid functions. However, we are only begining to understand its role in myeloid development [7,30]. The absence of information regarding the role of Ikaros in the myeloid context led us evaluate differences and similarities between these two groups; reorganizing our original pathologies and forming two new groups: lymphoid (B-ALL, CLL and MM) and myeloid (AML and CML).

**Figure 3:**
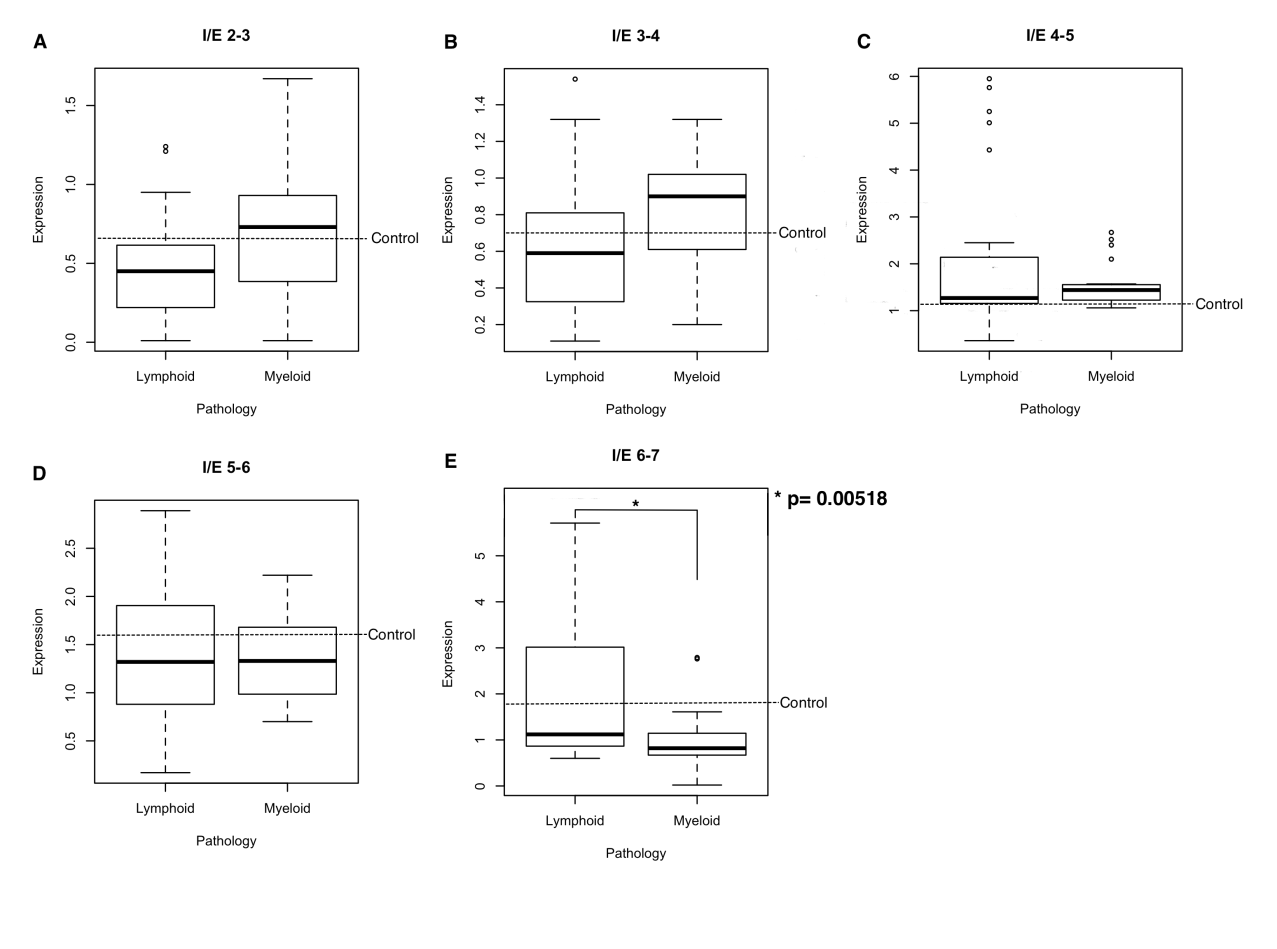
Level of expression of every interexon in Lymphoid vs Myeloid pathologies. (A) expression of the interexon 2-3, (B) expression of the interexon 3-4, (C) expression of interexon 45, (D) expression of the inter-exon 56 and (E) expression of interexon 6-7.

Data were analyzed using the same statistics process presented in the groups comparison and the results of the ANOVA showed a p value > 0.05, indicating that there are no significant differences for these two groups in the interexons 2-3, 3-4, 4-5 and 5-6. However, when we performed the ANOVA analysis for interexon 6-7, a p value of 0.01 was obtained and the Tukey statistical p value was 0.00518 (Figure 3). This result indicates that there may be a differential expression of isoforms with functional carboxyl terminal ends between lymphoid and myeloid diseases, possibly associated with differences in function in each case.

#### Changes in the Expression of the Ikaros Isoforms in the Evolution of a Progressive Case of CML to B-ALL

**Figure 4:**
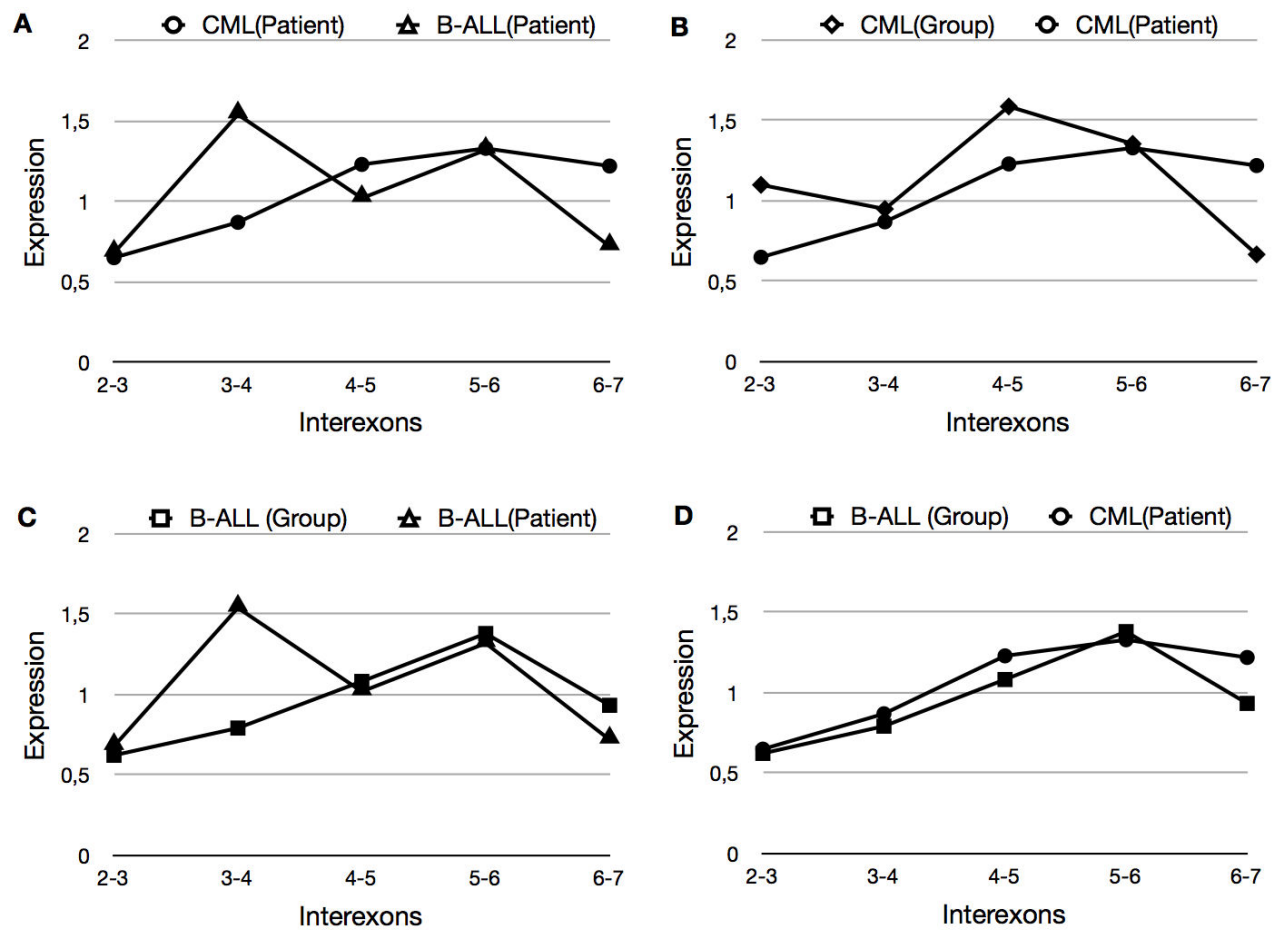
Ikaros perfil in a case of blastic crisis. (A) Levels of expression of the patient in B-ALL vs CML, (B) Comparison between the levels of expression of the CML group vs. expression of the patient with progressive pathology in CML stage, (C) Comparison between the levels of expression of the B-ALL group vs. expression of the patient with progressive pathology in B-ALL stage.

There are CML cases which can evolve into a lymphoid neoplasia; this is due to a change at the phenotypic level of tumor cells and it is hoped that this change will also be reflected in the levels of expression of the Ikaros isoforms. In this study, we had a patient who was characterized at pathology level and initially diagnosed as CML. Later, the patient showed a progression from the initial pathology, and was diagnosed as B-ALL. Thus, we were able to assess the change of expression of the Ikaros interexons in the same patient at two different times of disease progression (Figure 4).

As expected, the Ikaros expression at every moment and in every disease profile is easily distinguishable and shows that the expression of Ikaros isoforms varies depending on the pathology (Figure 4A). However, more interesting than this foreseeable difference is seeing that the pattern of expression of each phase of the disease does not match the profile of the condition in which the patient was diagnosed. This could indicate a difference at the molecular level between the novo pathologies and progressive pathologies (Figure 4B and 4C). With these data, note the curious similarities found between the patterns of Ikaros in the first stage of the disease and the typical pattern characterized by the B-ALL group, which the patient later developed (Figure 4D). Along with the change of expression expected in the evolution of the disease, the patient developed non-canonical splicing changes in interexon 4-5.

## Discussion

### New paradigm and new uses of the Ikaros family

In recent years, many of the new discoveries on role of the Ikaros family in the development of hematological cancers were related to dominant negative isoforms and their association with the development of specific pathologies. However, the lack of quantitative data as well as the presence of these dominant negative isoforms in normal cells from healthy individuals has limited our understanding of their role in pathology. It is likely that the maintenance of a delicate balance in the expression levels of these isoforms is essential for proper cell function and how this balance is disrupted defines the disease. Therefore, a pathological behavior may be directly related to the break of the balance in the levels of different isoforms. Alterations in Ikaros isoforms expression levels could be therefore considered as a player in the development of cancer [31-34].

 The technical complexity for analyzing and detecting the expression of these isoforms has prevented, in part, their use as biomarkers. In this paper, we propose a shift in the paradigm that allows us to dissociate the idea of isoform in pursuit of a real quantification of expression of the gene in the cell. In other words, our method allows us to create a profile for each part of the gene expressed, regardless of the existence of a concrete set of isoforms. This change of concept is necessary for a more accurate quantification of the gene. 

As the 'mutator phenotype' can be considered the reflection of a defect in the DNA repair systems [35], it is quite possible that the basis of the aberrant expression in splicing systems and the imbalance of Ikaros isoforms is another consequence of this process. The causes of this failure could be alterations in the spliceosome, but also be due to epigenetic alterations in the exonic regions that have been lately associated with the choice of the exons [36].

The first attempt aiming at a quantification of the Ikaros levels was carried out by Iacobucci et al. in 2008. This study allowed the authors to obtain quantifiable peaks of known isoforms. However, the inability to identify certain peaks and the abundance of isoforms in every gene in this family means that this system has conceptual gaps which are difficult to solve. It is a good system to quantitate known isoforms, but not to determine the total number of isoforms in the cell. Taking this into account, in this paper we present a system of quantification for the Ikaros family based on the measurement of each exon separately, regardless of the isoform concept. Thus, the most innovative work relates to the design of the PCRs. This measurement is made by two PCRs per exon because the design of primers was thought to amplify interexonic regions. This design has certain benefits. First, the information related to each exon depends on two PCRs and avoids the amplification of genomic regions. In addition, this design allows us to use the melting curve to extract more information such as the presence of non-canonical splicing, which is common in certain exons, for example, insertions and deletions in Ikaros or the alternative exons and splicing in other members of the family such as Helios and Aiolos [10,37]

One of the first findings from observing the expression of Ikaros in different diseases, as predicted by the literature, is that in these types of pathologies there is a high expression of DN isoforms. This is shown by the low expression levels observed for interexons 2-3, 3-4 and 4-5, which are regions that encode for the DNA-binding domain. This is very clear in the cases of B-ALL, CLL and AML (Figure 1A, 1B and 1C). For example, similar results appear in the work of Iacobucci and colleagues [25], which confirms the over-expression of the DN Ik-6 isoforms, which loses its full DBD in patients of B-ALL Philadelphia positives. 

 Also, that work confirms the existence of gene variants represented by insertions of 60 bp upstream of exon 2 and deletions of 30bp at exon 6 end in several Ikaros isoforms [25]. Similarly, our study shows that some kind of change in the splicing occurs in region 4-5 and 6-7, possibly derived from deletions, taking into account the change of the melting Temperature.  The non-canonical splicing in IE/4-5 is a typical brand of B-ALL, appearing in more than 85% of cases, while in the case of the interexon 6-7, although it is not so frequent, it seems that it is an exclusive brand of this pathology, and it can be divided into two subgroups this pathology, which may be important in the outcome of the disease.

 Iacobucci conclude that the over-expression of the Ik-6 isoform coupled with the emergence of anomalies at the level of sequence are common features in cases of B-ALL Philadelphia chromosome positive (BCR-ABL1) [25]. In a similar way, our method allows us to conclude that this type of leukemia is characterized by the presence of dominant negatives and non-canonical splicing in the interexon 4-5 and 6-7. However, according to the pathological samples, these alterations in splicing are not associated with the presence of Philadelphia chromosome, since none of the patients with non-canonical splicing presented the translocation.

 The pattern of expression presented by patients with multiple myeloma shows differences in expression that are more irregular than any of the other four groups evaluated, showing low expression in regions 2-3, 3-4 and 5-6 and an over-expression in the exons 4-5 and 6-7 (Figure 1 and 2). These overexpressed regions are essential for the structure of the Ikaros protein. Exon 4 encodes two of the four zinc fingers that compose the DBD and exon 7 encodes two zinc fingers that compose the dimerization domain, which suggests that this disease produces functional isoforms with the presence of the DBD and/or the dimerization domain. We could even assume, taking into account the distribution of data, that there are isoforms Δ5 and Δ6, but not Δ5, 6 in the MM. This suggests that there should be other isoforms or other genes and other transcription factors that are altered. In any case, the three interexon 4-5, 5-6 and 6-7, allow us to differentiate this condition from the others with a single PCR, which is one of the main results of this study. The same finding applies to the CLL: with the results of the interexon 5-6 and 6-7 we can differentiate from the other studied pathologies. These results indicate that this methodology could serve, in the future, for the diagnosis of these two diseases through a very simple single PCR. Although it is clear that the diagnosis of these diseases it is perfectly standardized with other techniques, none of these use Ikaros as a biomarker. Our data indicates that the analysis of Ikaros family isoforms could provide additional information. For instance, these analyses could be useful for prognosis and response to treatment. In this sense, a long-term study is necessary to check the possible implications on prognosis and evolution of the disease. It may also be interesting in diseases that do not currently have a method clear to their prognosis, such as gammopathies of uncertain meaning that evolve to MM. Taking into account the discriminatory ability of the method for the MM, it could be useful in diagnosing the early evolving disease, that at this time only you can know after the evolution and not before. 

 There is a correlation between genetic and morphological alterations in bone marrow and peripheral blood in any type of hematological diseases (except MM) that can be tested to see the profile of healthy controls against the pathologies. Thus, the controls always had a range in the expression levels similar to the group that did not show differences. In other words, we cannot differentiate controls against acute leukemia, but we can differentiate them from MM and CLL, and this is the interesting aspect for the characterization of these two pathologies.

Some of the pathologies can easily be discriminated with a single PCR. It is very important to improve discrimination by creating a profile that contains all possible information of the expression of Ikaros. For this reason, an analysis of clustering that allowed to obtain a more accurate profile of each disease. In this study, it was taken into account both, the levels of expression of each interexon and the presence of not canonical splicing, which seem to be very specific for certain pathologies. This set of data allowed us to separate four groups of diseases with more differentiated features and allows to observe profiles very unique to certain groups, such as MM and CLL (Figure 2A). Thus, the expression of the interexon 6-7 does not allow us to differentiate B-ALL from AML or CML, but if we take into account the presence of non-canonical splicing, we can distinguish a group with a double peak in interexon 6-7, that are unequivocally B-ALL (Figure 2). These results make it clear that the isoforms present in every type of disease are different and define the disease, which clearly demonstrates the potential of this family of genes in neoplastic hematologic characterization.

AML analysis, such as expected by ANOVA, did not results in the ability to define a group suitable for this disease. It is possible that for this disease in particular, data provided by other members of the family could contribute to a more precise characterization; therefore, other family members, such as Aiolos and Helios, need to be analyzed. This analysis would provide more information to help increase the power of discrimination between groups of pathologies. Interestingly, when analyzing the cluster for this disease, it is possible to observe two subgroups of AML, one that is similar to the CML group and a second group more close to CLL (Figure 2B). However, no clinical, pathological or immunophenotypic characteristics can explain this division. Both groups are varied and of different natures (monocytic, granulocytic or promyelocitic).

### Differences between Lymphoid and Myeloid Neoplasms

Ikaros is an important regulator of gene expression and plays a very important role in differentiation, proliferation and function of lymphoid cells [38,39]. However, its role at myeloid level has only started to be studied. The Ikaros isoforms that appear in this lineage may be related to cellular regulation and commitment in mice and humans [7,30]. Because of the absence of information on the role of Ikaros in myeloid and myelo-erythroid development, we aimed at evaluating the differences and similarities between both types of pathologies.

We reorganized our groups of pathologies and formed the group of lymphoid diseases (B-ALL, CLL and MM) and myeloid diseases (AML and CML). The results show that there are significant differences between these two groups of pathologies. This result suggests that the Ikaros isoforms present in lymphoid leukemia are different from those in the myeloid group. Failing to learn more about their role in myeloid lines, it is feasible to assume that there are differences in the progression of the disease, possibly due to differences in the tissue’s natural expression, i.e., that the differences noted in pathologic states may be caused by functional differences that the Ikaros gene may present in the myeloid lineage. 

### Molecular Nature of Progressive CML

As it has been commented on in the results, there are cases in which a patient diagnosed with CML can evolve to blastic crisis lymphoid (ALL) or myeloid (AML). Currently, these cases are rare, especially with current tyrosine kinase inhibitors treatment that modifies the evolution of the disease in its very early stages. In this work, we included a case in which the patient evolved from CML to B-ALL, that allowed us to compare how Ikaros expression varied in parallel to the evolution of the disease. The results show that each stage of the disease has its own profile of the Ikaros gene expression (Figure 4A). Apart from this expected change, both stages of the disease also differ from the rest of the patients for each pathology (Figure 4B-4C). This finding is extremely important because it suggests that while by pathological and immunophenotypical methods can easily group this patient into CML or B-ALL at any moment, there are molecular differences in the expression of Ikaros that suggest more subtle differences in this case. These data indicate the existence of molecular differences that evolve in CML blast crisis, which would indicate two different pathologies at molecular level. Thus, together with the differential value, which has proven this gene for the definition of the disease, the Ikaros gene could be a terrific prognostic factor that would allow us to distinguish between patients who will evolve to blastic crisis and those who will not. It is of note the similarity between the pattern of expression in the first stage of the disease with respect to the one observed in the disease that will later evolve (Figure 4D). This could mean that we could predict not only the evolution, but also the nature of this evolution.

## Supporting Information

Figure S1 **Level of expression of every interexon (I/E) in all pathologies, with AML divided in two groups.** (A) expression of the inter-exon 2-3, (B) expression of the inter-exon 3-4, (C) expression of inter-exon 4-5, (D) expression of the inter-exon 5-6 and (E) expression of inter-exon 6-7. AML1 Shows the acute Myeloid Leukemia group similar to the CML, while AML2 is the group that shows a Ikaros profile closer to the CLL. CML (chronic myeloid leukaemia), MM (multiple myeloma), CLL (chronic lymphoid leukemia), BALL ( B acute lymphoblastic leukemia).
(TIFF)

Figure S2 **Profile of Ikaros comparison between CML, CLL and the two groups of AML observed by clustering.** The graphic represents the percentage of each interexon respect to the total.
(TIFF)
