# Supplementary figures and images for: The Combined Expression Patterns of Ikaros Isoforms Characterize Different Hematological Tumor Subtypes
Source: PLoS One. 2013 Dec 6;8(12):e82411. doi: 10.1371/journal.pone.0082411 (PMC3855751; doi:10.1371/journal.pone.0082411)

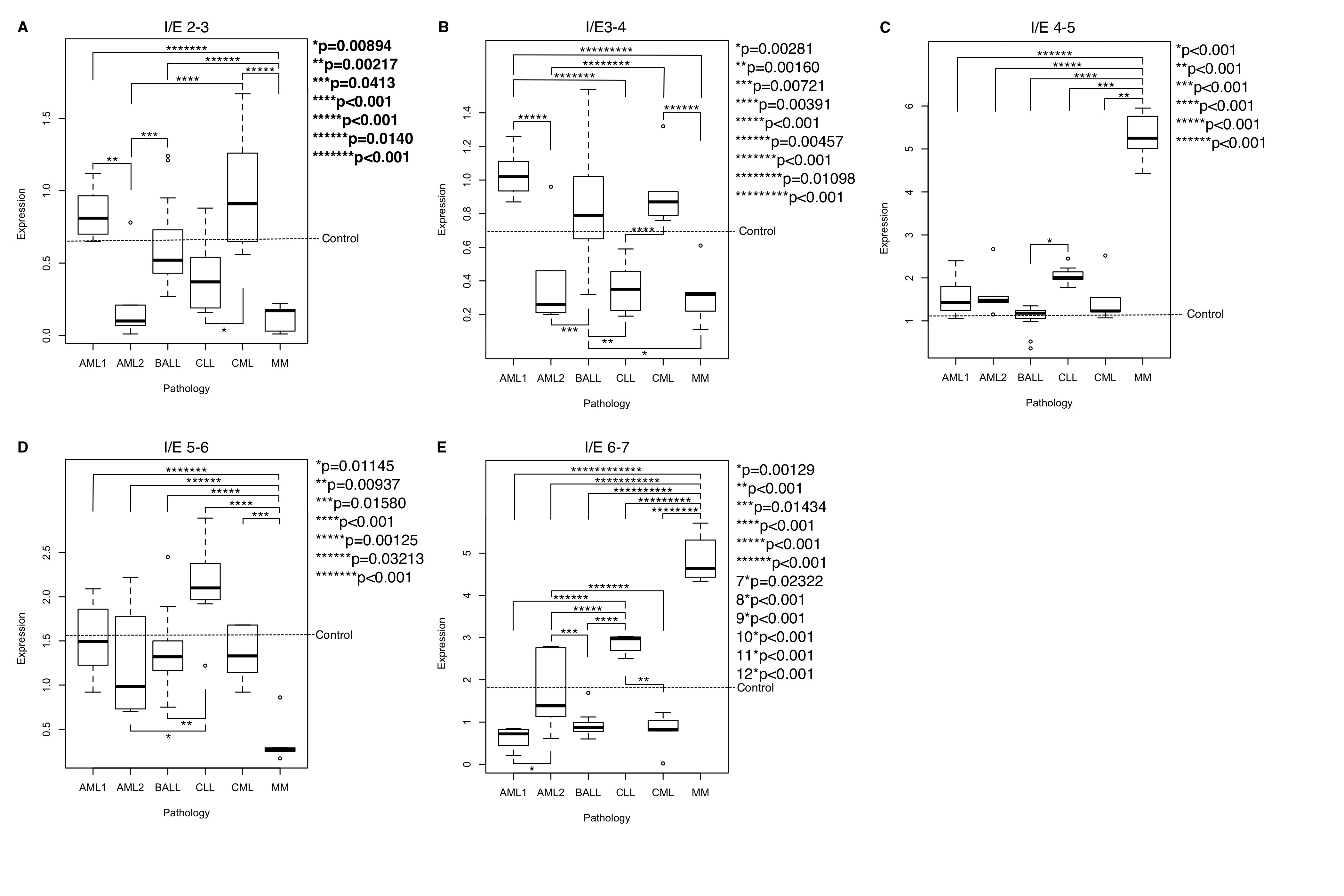

Supplement: Figure S1 — Level of expression of every interexon (I/E) in all pathologies, with AML divided in two groups. (A) expression of the inter-exon 2-3, (B) expression of the inter-exon 3-4, (C) expression of inter-exon 4-5, (D) expression of the inter-exon 5-6 and (E) expression of inter-exon 6-7. AML1 Shows the acute Myeloid Leukemia group similar to the CML, while AML2 is the group that shows a Ikaros profile closer to the CLL. CML (chronic myeloid leukaemia), MM (multiple myeloma), CLL (chronic lymphoid leukemia), BALL ( B acute lymphoblastic leukemia). (TIFF) [file pone.0082411.s001.tif]

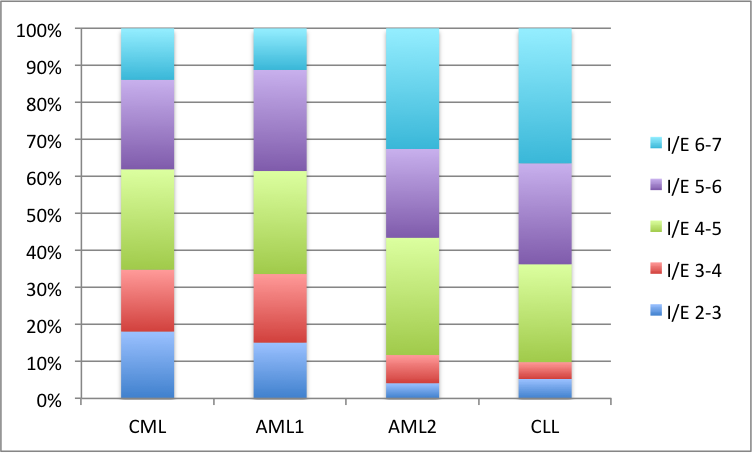

Supplement: Figure S2 — Profile of Ikaros comparison between CML, CLL and the two groups of AML observed by clustering. The graphic represents the percentage of each interexon respect to the total. (TIFF) [file pone.0082411.s002.tiff]
